# Phenotypic variation between siblings with Metachromatic Leukodystrophy

**DOI:** 10.1186/s13023-019-1113-6

**Published:** 2019-06-11

**Authors:** Saskia Elgün, Jakob Waibel, Christiane Kehrer, Diane van Rappard, Judith Böhringer, Stefanie Beck-Wödl, Jennifer Just, Ludger Schöls, Nicole Wolf, Ingeborg Krägeloh-Mann, Samuel Groeschel

**Affiliations:** 1 Department of Paediatric Neurology and Developmental Medicine, University Children’s Hospital Tübingen, Hoppe-Seyler-Strasse 1, 72076 Tübingen, Germany; 2 Department of Child Neurology, Emma Children’s Hospital, Amsterdam University Medical Centers, Vrije Universiteit Amsterdam, and Amsterdam Neuroscience, Amsterdam, The Netherlands; 3 Department of Medical Genetics, University Hospital Tübingen, Tübingen, Germany; 4 Clinical Neurogenetics Section, Department of Neurology and Hertie Institute for Clinical Brain Research, University of Tübingen, Tübingen, Germany; 5 German Center for Neurodegenerative Diseases (DZNE) Tübingen, Tübingen, Germany

**Keywords:** Metachromatic leukodystrophy, Siblings, Genotype, Natural course, Genetics

## Abstract

**Background:**

Metachromatic Leukodystrophy (MLD) is a rare autosomal-recessive lysosomal storage disorder caused by mutations in the *ARSA* gene. While interventional trials often use untreated siblings as controls, the genotype-phenotype correlation is only partly understood, and the variability of the clinical course between siblings is unclear with some evidence for a discrepant clinical course in juvenile patients. The aim of this study was to systematically investigate the phenotypic variation in MLD siblings in comparison to the variability in a larger MLD cohort and to case reports published in literature.

**Results:**

Detailed clinical information was available from 12 sibling-pairs (3 late-infantile, 9 juvenile) and 61 single patients (29 late-infantile, 32 juvenile). Variability of age at onset was similar between the siblings and randomly chosen pairs of the remaining cohort (no statistically different Euclidean distances). However, in children with juvenile MLD both the type of first symptoms and the dynamic of the disease were less variable between siblings compared to the general cohort. In late-infantile patients, type of first symptoms and dynamic of disease were similarly homogeneous between siblings and the whole MLD cohort. Thirteen published case reports of families with affected siblings with MLD are presented with similar findings.

**Conclusions:**

In a systematic analysis of phenotypic variation in families with MLD, siblings with the late-infantile form showed a similar variability as unrelated pairs of children with late-infantile MLD, whereas siblings with juvenile MLD showed a more homogeneous phenotype regarding type of first symptoms and disease evolution in comparison to unrelated children with juvenile MLD, but not regarding their age at onset. These results are highly relevant with respect to the evaluation of treatment effects and for counseling of families with affected siblings.

## Background

Metachromatic Leukodystrophy (MLD) is an autosomal recessive, monogenic disease caused by mutations in the arylsulfatase A (*ARSA*) gene, leading to deficiency of the enzyme *ARSA* and therefore inadequate degradation of sulfatides [1, 2]. Sulfatides accumulate especially in the central and peripheral nervous system, and lead to progressive demyelination and neurological symptoms [1, 2]. The clinical course can be divided into a pre-symptomatic stage with normal development, followed by onset of first symptoms and a period of developmental stagnation. This plateau phase is shorter in early onset forms, and longer and more variable in late onset forms. Finally, rapid disease progression evolves with a relatively invariable rapid loss of gross motor function, and a final stabilization at a low functional level [3].

Genotype-phenotype correlation revealed that null alleles, which cause hardly any residual *ARSA* activity [4, 5], result in an early onset and rapid deterioration of motor and cognitive function characterizing the late-infantile form of MLD with first symptoms occurring before 2.5 years of age [6]. In later onset forms (juvenile MLD with disease onset between 2.5 and 16 years, and adult MLD with disease onset after 16 years of age), the prevalent genotypes were associated with some remaining residual activity of the enzyme [4, 5]. Although this allows some genotype-phenotype correlation, the exact relationship between genotype, residual enzyme activity, and clinical phenotype remains to be elucidated. Today more than 250 *ARSA* mutations are known, making it challenging to define more precise genotype-phenotype relationships especially in the later onset forms, which often show compound heterozygosity for different mutations [7–11].

The phenotypic variability becomes especially relevant when treatment evaluation is based on comparison with an untreated sibling carrying the same mutations [12, 13]. Younger siblings of an affected older sibling are usually diagnosed in an early (or even pre-symptomatic) disease stage and have the opportunity to undergo a treatment like conventional or gene-corrected stem cell transplantation [14–17]. However, it is unclear how their clinical course would have evolved without treatment. Case reports suggest that there might be some variability of disease onset even between siblings with MLD [18, 19], but a systematic analysis is currently missing.

The aim of this study is to systematically investigate phenotypic variability in a significant number of siblings with MLD, regarding disease onset and progression, and compare them to the phenotypic variability in a large cohort of children with MLD. In addition, case reports in the literature will be discussed.

The results are not only relevant for counseling of families with affected children, but will also allow better interpretation of treatment outcomes, and might help to better understand the complex genotype-phenotype relationships in MLD.

## Results

### MLD siblings and cohort

From our database 85 children with late-infantile (*n* = 29) and juvenile (*n* = 56) MLD fulfilled inclusion and exclusion criteria for this study (see Methods). From these, 12 sibling pairs (24 patients) were identified (three late-infantile, nine juvenile), three of them from the Center for Children with White Matter Disorders, VU University Medical Center, Amsterdam. Three of the juvenile sibling pairs were analyzed only regarding the onset of symptoms, as at least one of them received a therapeutic intervention potentially influencing the further course of disease. Table 1 gives an overview of the clinical and diagnostic findings of all sibling pairs.

**Table 1:** Analyzed siblings with MLD

Sibling pairs		Gender	MLD-form	Age at onset, years	Type of onset	MRI-score, early stage	Genotype
1	.1 .2	m f	late-infantile	1.8^a^ 2.1	motor	16 16	c.575C > G; c.733G > A^b^
2	.1	m	late-infantile	1.5^a^	motor	–	–
	.2	f		1.5		–	
3	.1	f	late-infantile	0.8^a^	motor	20	c.449C > T; c.449C > T
	.2	f		1.8		20	
4	.1	m	juvenile	11^a^	cognitive	20	c.542 T > G; c.1468 T > C
	.2	f		12.4		20	
5	.1	f	juvenile	5.6^a^	mixed	–	–
	.2	f		6.7		–	
6	.1	f	juvenile	6.2^a^	motor	15	c.465 + 1G > A; c.1283C > T
	.2	f		4.7		14	
7	.1	f	juvenile	14^a^	mixed		c.1283C > T; c.1283C > T
	.2	f		11.9		15	
8	.1	m	juvenile	6.5^a^	cognitive	18	c.465 + 1G > A; c.542 T > G
	.2	m		13.5		17	
9	.1	m	juvenile	10^a^	motor	–	c.1283C > T; c.1283C > T
	.2	f		15		–	
10	.1	f	juvenile	8	mixed	19	c.465 + 1G > A; c.1283C > T
	.2	m		5		20	
11	.1	m	juvenile	6.7	motor	22	–
	.2	f		6		21	
12	.1	m	juvenile	5	motor	20	–
	.2	m		5		20	

^a^ = first diagnosed sibling (if known); ^b^: genotype in traditional nomenclature

### Age at onset of clinical symptoms

**Fig. 1:**
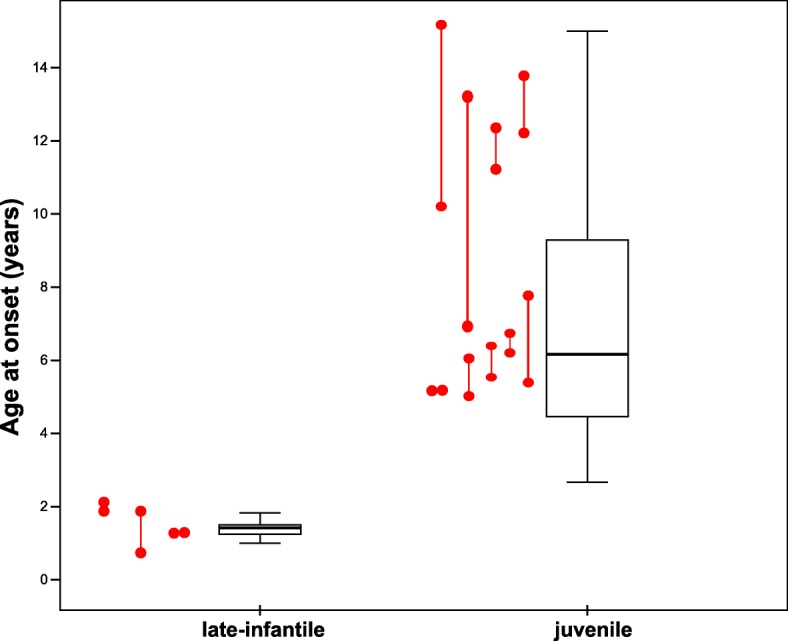
Age at onset of the cohort (late-infantile / juvenile). Box plots indicate distribution of age at onset for non-related children with MLD. Siblings are marked in red, with lines connecting pairs of siblings

As shown in Fig. 1, siblings with MLD showed a similar variability with respect to age of onset of first symptoms compared to the rest of the MLD cohort. Although some sibling pairs were close in their age at onset, others showed a relevant discrepancy. One sibling pair with late infantile MLD had a difference of 1 year in age at onset (with the later onset in the second sibling). In juvenile MLD, 7 out of 9 sibling pairs with juvenile MLD had an onset more than 1 year apart, four more than 2 years, and in one sibling pair age at onset differed more than 6 years. Although the variability of the age at onset between unrelated pairs of the juvenile cohort was higher on average (mean Euclidean distance 44.37 months, standard deviation (SD) 33.99) compared to the sibling pairs (29 months, SD 27.05), this difference was not statistically significant (*p* = 0.16). This was true also for the unrelated late-infantile cohort with a mean Euclidean distance of the age at onset of 4.4 months (SD 3.57) vs. 5.33 months (SD 6.11) in sibling pairs (*p* = 0.91).

### Symptom constellation at onset

When classifying symptom constellations at the age at onset into gross motor, cognitive symptoms or both, all examined sibling pairs showed the same symptom constellation at onset, even when their age of onset was different. While the children with late-infantile MLD all had motor symptoms at onset, the children with juvenile MLD presented with all three categories as shown in Table 1. In the unrelated group of children with juvenile MLD 14 had motor onset, 17 had cognitive onset and 7 had mixed onset.

For each of the three categories, one example is presented:

The older sibling of pair 1 (cf. Table 1) presented with gait difficulties, abnormal movement patterns and weakness at the age of 21 months (after having learned to walk independently aged 12 months). His sister presented with the same first motor symptoms at the age of 25 months (independent walking from age 14 months). Both siblings deteriorated rapidly, with complete loss of gross motor function in the months following due to severe progressive spastic tetraparesis.

Sibling 8.1 was noticed with concentration difficulties at the age of 6 years (78 months) and described with slowly declining cognitive abilities. As a result his school grades deteriorated, especially in mathematics, and he had to repeat a year in school and eventually change school type. On the other hand, his motor function remained unaffected into adulthood. His younger brother (8.2) started to develop very similar school problems at the age of 13 years (162 month), subsequently he had to repeat a year in school and then changed type of school. In addition, he had problems in his social behavior at disease onset with alcohol abuse, aggressive behavior and a suicide attempt. Like in his older brother, his motor function remained stable into adulthood.

Sibling pair 5 presented with both cognitive and motor symptoms at onset. At the age of 5 years, patient 5.1 showed a whiny mood and developed toe gait. Patient 5.2 also presented with whiny mood, orientation problems, and loss of previously acquired anorectal continence at the age of 6 years, quickly followed by gait difficulties.

### Disease dynamics

#### GMFC-MLD

**Fig. 2:**
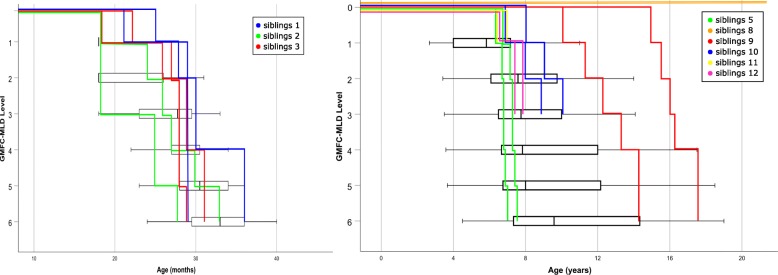
Disease progression (of gross motor function). Progression of gross motor function (GMFC-MLD levels 1 to 6) of children with late-infantile (left) and juvenile (right) MLD; box plots shows distribution of non-related children with MLD within each level, siblings are marked by colored lines; note: GMFC-MLD is only applicable after the age of 18 months (=90th percentile of walking) [3]

In Fig. 2 progression of gross motor function of the different pairs of siblings is shown in relation to the rest of the whole (unrelated) cohort. Motor function was assessed using the standardized gross motor function classification for MLD (GMFC-MLD) [20]. The figure illustrates that siblings, regardless of their age of onset, have a similar disease progression. In the late-infantile group (Fig. 2, left), all children, whether siblings or not, showed a rather uniform and rapid progression of their motor function. In the juvenile group, the variability of the duration from GMFC-MLD level 1 (age when first motor symptoms were noticed) to GMFC-MLD level 3 (losing the ability to walk) was lower between sibling pairs than between non-related pairs of the juvenile cohort (7.00 months (SD 6.16) versus 23.66 months (SD 20.35), Fig. 2, right). This was, however, not statistically significant, *p* = 0.077.

#### Cognitive disease progression parameters

For progression of cognitive symptoms, we investigated the group of children with juvenile MLD only, as the late-infantile group showed a very rapid and uniform disease progression dominated by the loss of gross motor function, but also including the loss of their cognitive abilities [3, 21].

Between siblings with juvenile MLD, the variability in time from first symptoms to presenting with concentration problems was smaller (2.67 months, SD 4.62) compared to unrelated pairs of the rest of the juvenile MLD cohort (19.72 months, SD 14.46, *p* = 0.015). The variability when first language decline started, however, was similar between pairs of siblings (mean Euclidian distance 29.00 months, SD 37.58) compared to unrelated pairs of the juvenile MLD cohort (mean 29.01 months, SD 25.08).

#### MRI

**Fig. 3:**
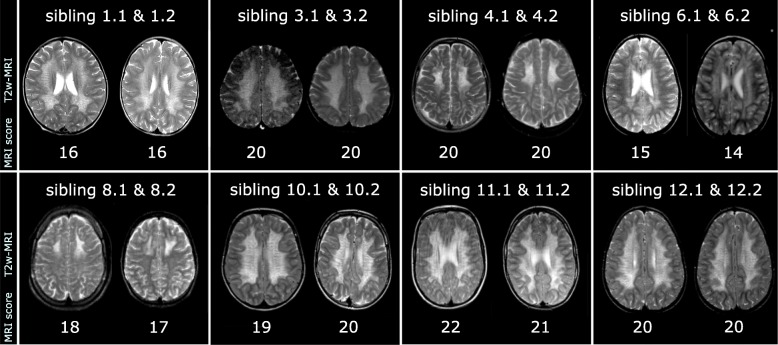
T2-weighted MRI of sibling pairs in early disease stage. MRI of sibling pairs in early disease stage (at diagnosis) with the respective MRI score below illustrating the similarity in distribution of white matter changes between siblings. Axial slices of T2-weighted sequences were selected

Table 1 and Fig. 3 show MRI scores and MR images of siblings with MLD. Both the scores and pattern of demyelination appeared very similar between the siblings. Especially in children with juvenile MLD, frontal or parieto-occipital involvement was always similar, and was related to a more cognitive or motor symptom onset.

#### Genotype

In order to analyze the genotype-phenotype relationship, we identified the most common genotypes and compared the variability of age at symptom onset between siblings and non-related children carrying the same genotype.

**Fig. 4:**
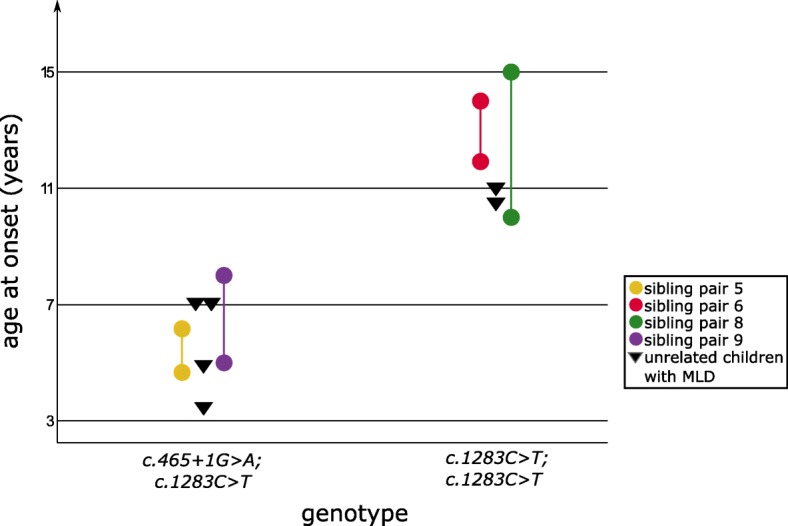
Genotype – Age at onset. Variability of age at symptom onset between siblings and non-related children, carrying the same genotype, in two most common genotypes

The results, displayed in Fig. 4, show that the age at onset in the patients with compound heterozygosity for c.465 + 1G > A and c.1283C > T in *ARSA* gene ranged between 3.5 and 8 years (mean 5.8 years of age; *n* = 8). Homozygosity for c.1283C > T caused a later disease onset with a range between 10 and 15 years (mean = 12.1 years; *n* = 6). Siblings with these genotypes were within the range of unrelated children in both genotypes without being more similar to each other. Therefore, the age of onset between siblings with MLD was not more similar compared to non-related children with MLD carrying the same genotype.

## Discussion

MLD is an inherited disease with an autosomal recessive trait, sometimes affecting several siblings in one family. With regard of the natural disease course in siblings, there exist only case reports. Some of them indicate a very similar clinical course and some show more variability between the siblings. This study represents the first systematic analysis of the phenotypic variability in siblings with MLD.

We were able to show that age of onset may vary considerably between siblings with MLD. Although some sibling pairs share a very similar age of onset, others show a high discrepancy. Within the same family, however, the onset and course is either late infantile or juvenile. Even within late infantile MLD onset in siblings may be up to 12 months apart. In juvenile MLD, around 80% have a disease onset of more than 1 year apart and around 40% more than 2 years. This variability in age of onset is similar between the siblings and between randomly chosen unrelated pairs of the MLD cohort.

Case reports of siblings in the literature, summarized in Table 2, also show this variability. The first description of siblings with MLD was reported by Scholz in 1925 [22]. He described two siblings with the juvenile form and a quite similar age of disease onset at around 8 years of age, both with cognitive and motor symptoms at onset (brother 1: spasticity in legs resulting in rigid and slow walking, optical atrophy, learning and perception problems as well as emotional lability; brother 2: spasticity in extremities, tremor in legs, whiny, unstable mood and cognitive problems (learning, perception)) and a rapidly progressive disease course, both died around 3 years after onset in a very advanced disease stage. Alves et al. [23] reported on four cases of late onset (15, 17, 18, 21 years) MLD in a family with 15 siblings. In accordance with our results the described siblings all had the same symptom cluster at onset - mental deterioration and/or behavioral changes. A quite similar case by Satoh et al. [24] reported on a sibling pair with motor and cognitive symptoms at disease onset at the ages of 19 and 15 years. Koul et al. [25] described two affected siblings with late-infantile MLD both with an onset with deterioration of motor function at 2 years of age. Mahmood et al. reported triplets with the late-infantile form, all of them with motor function starting around 16 months of age after normal psychomotor development [26]. Another report of siblings with late-infantile MLD by Nyberg-Hansen et al. [27] delineated 2 brothers, who both developed motor dysfunction and dysarthric speech within the second year of life with rapid progression in the following years. Aslan et al. [28] reported on two siblings with the juvenile form of MLD, who had their first symptoms at 6 and 7 years of age respectively, also showing a similar symptom constellation at onset with both motor and cognitive signs (the first sibling: ataxia, attention deficit and perceptual difficulties; the second: learning difficulties, mild changes in fine and gross motor function). The later onset case reports by Hoes et al. [29], Cengiz et al. [30] and Manowitz et al. [31] also have in common a similar symptom constellation at onset (Table 2). Hoes et al. reported on two brothers: the first developed first symptoms at the age of 27 years with problems in social interaction soon followed by hypersexual, aggressive and apathetic behavior, he died at the age of 30 years after a kidney biopsy [29]. His younger brother showed first symptoms at the age of 26 (social handicap, psychiatric symptoms) and died within 8 years after onset [29]. Cengiz et al. described three sibling with variability in their age at onset of clinical symptoms (21, 18 and 12 years) but very similar symptom constellation (1: behavioral disturbance, working memory difficulties, epileptic seizures; 2: epileptic seizures and progressive mental changes; 3: poor school performance, disinhibition, hyperactivity and self-awareness) [30]. Furthermore Manowitz et al. described an adult sibling pair with cognitive symptoms at onset and late initiating motor progression [31]. These case reports corroborate our findings by showing some variability in age of onset (maximum 9 years difference in Cengiz et al.), but clear similarity in the type of first symptoms and disease progression (e.g. Scholz 1925: rapid, motor and cognitive, died within a few years; Alves: pronounced severe mental deterioration in disease course). This is especially remarkable as children with juvenile MLD are known to show a heterogeneous presentation and clinical course. One report from 1981 by Yatziv and Russell et al. [32], was difficult to interpret. They described a family with three affected siblings; the eldest showed first symptoms (developmental delay) aged around 1 year, while the two younger siblings were reported with a disease onset at 6 years, with all three developing dystonia as their dominant sign with normal cognitive function into adulthood. As none of the other symptoms usually reported in MLD were described [3, 21] and the diagnosis was based on enzyme measurements, where pseudodeficiency and carrier status for pathogenic mutations may explain relatively low values, diagnosis of MLD seemed uncertain, and we did not include this report in Table 2.

**Table 2:** Case reports of siblings with MLD

Author	Year	Sibling	Age at onset (years)	Type of onset	Genotype
Scholz	1925	1	8	cognitive and motor	–
		2	8	cognitive and motor	–
Nyberg-H.	1972	1	1	motor	–
		2	2	motor	–
Hoes	1978	1	27	cognitive	–
		2	26	cognitive	–
Manowitz	1978	1	16	cognitive	–
		2	18	cognitive	–
Alves	1986	1	18	cognitive	–
		2	21	cognitive	–
		3	17	cognitive	–
		4	15	cognitive	–
Satoh	1988	1	19	cognitive and motor	–
		2	15	cognitive and motor	–
Clarke	1989	1	9	cognitive	–
		2	23	pre-symptomatic	–
Kappler	1992	1	14	cognitive	p.R84Q; p.P426L
		2	29	pre-symptomatic	p.R84Q; p.P426L
Koul	1994	1	2	motor	–
		2	2	motor	–
Arbour	2000	1	7	cognitive + seizures	p.W318ter; p.R143G
		2	22	cognitive	p.W318ter; p.R143G
Cengiz	2002	1	21	cognitive + seizures	–
		2	18	cognitive + seizures	–
		3	12	cognitive	–
Mahmood	2010	1	1.3	motor	c.459 + 1G > A; c.459 + 1G > A
		2	1.3	motor	c.459 + 1G > A; c.459 + 1G > A
		3	1.3	motor	c.459 + 1G > A; c.459 + 1G > A
Aslan	2018	1	7	cognitive and motor	c.1055 T > C; c.991G > A
		2	6	cognitive and motor	c.1055 T > C; c.991G > A

Apart from this case report, siblings of children with late-infantile MLD all had the late-infantile form with the typical rapid disease progression. It may not surprise that within the later onset forms, with a higher residual enzyme activity than in the late-infantile form, the age of onset can vary considerably. For example, Arbour et al. 2000 [19] reported on a Vietnamese family with two affected siblings, both with the same genotype, of whom the first was earlier and more severely affected starting with cognitive symptoms and epileptic seizures at the age of 7 years with a more rapid disease progression, whereas the second sibling had very mild cognitive symptoms at the age of 22 years. Another sibling pair was described by Clarke et al. 1989 [18], with clinical manifestation of MLD at the age of 9 years with cognitive symptoms (learning and behavioral difficulties) for one affected sibling. The second affected sibling presented none of the common symptoms until the age of 23 years (age at description) except sulfatide accumulation in the gallbladder. Also the siblings with juvenile MLD reported by Kappler et al. in 1992 show this kind of difference in the age of onset, with the older one presenting with behavioral changes at the age of 14 years [33].

It can currently only be speculated that there must be unrecognized factors that influence the phenotypic variability beyond the genotype level. It is interesting to note that even when children with the same genotype are investigated, the siblings amongst them (who share a substantial part of their genome) do not have a more similar age of onset compared with non-related children. It is likely that there are other epigenetic, metabolic or unidentified factors which influence the onset of first symptoms of MLD in addition to the genotype. Our results underline, that although probably valid in many cases, comparing treated patients to their non-treated siblings, has to be done with caution. Comparison to a larger non-treated cohort of children with MLD might be a more valid approach [34]. This underlines the importance of natural history data as retrospective controls for treatment trials, in the absence of the feasibility of randomized placebo-controlled studies in rare progressive disorders, like MLD.

There are some limitations in respect to our findings. Most importantly, although being the most comprehensive sibling study, larger numbers of patients would be desirable. While in our study we were able to sustain a high data quality due to the same investigators and clinical standards, a multi-centric international collaboration will increase patient numbers and potentially confirm our results with more statistical power and certainty.

In addition, data on disease onset are often retrospective (as long as neonatal screening is not available), and analysis of clinical parameters can be challenging. For example, age of disease onset might be influenced by a certain parental interpretation and perception. We have, however, used clearly defined clinical parameters and have validated parent reported disease onset by medical records and phone interviews [3, 21]. Furthermore, we have not observed a tendency in recognizing earlier symptom onset in the second sibling when the older sibling was already diagnosed. This is underlined by the fact that in 5 of 12 sibling pairs the first diagnosed (older) sibling had a disease onset at an earlier age than the next (younger) sibling.

Symptom constellations at disease onset may be difficult to identify. Some symptoms might be harder to pinpoint at a specific age (e.g. concentration problems, which might slowly start or be unspecific), other symptoms might start shortly after the first. Therefore, we considered them to belong to the first symptoms even if they appeared a short time after. From a pathophysiological or biochemical perspective, symptom onset might be related to a supra-threshold level of progressive pathological changes, like sulfatide accumulation, demyelination, or axonal damage. Further information is needed from biochemical or MRI biomarkers in order to define such supra-physiological thresholds.

## Conclusions

In the most comprehensive study presented so far, we investigated the phenotypic variability of siblings with MLD compared to unrelated pairs of children with MLD. We found that siblings with MLD show a similar type of first symptoms, MRI pattern, and dynamic of disease progression. However, regarding the age of disease onset some sibling pairs show considerable variability. As genotype-phenotype-correlation was not higher in siblings than in unrelated children with the same genotype, this suggests that additional biochemical and epigenetic factors might influence the clinical phenotype.

These data are important for family counseling, but also essential for the evaluation of treatment trials, where untreated siblings are often used as controls.

## Methods

### Patients

Clinical and MRI data of children with late-infantile and juvenile MLD have been collected retrospectively within the German leukodystrophy network LEUKONET since 2006. The study was approved by the local ethics committees (401/2005 and 2012/098). Written informed consent was given by at least one caregiver.

In addition, data from siblings with MLD from a patient cohort of the Center for Children with White Matter Disorders, VU University Medical Center, Amsterdam, were included.

The diagnosis of MLD was made based on increased sulfatide excretion and ARSA deficiency in the context of clinical symptoms and MRI, in addition, confirmed by genetic analysis in most cases. Data after therapeutic intervention (e.g. stem cell transplantation) were excluded from the analysis in order to describe the natural disease course.

### Clinical parameters

The disease onset was defined as first neurological symptoms or loss of motor and/or cognitive skills. The type of first symptoms was classified into three categories: only motor symptoms, only cognitive symptoms or both, based on a questionnaire and verification by phone and/or medical records as done in Kehrer et al. 2011/2014 [3, 21]. Motor symptoms were defined as gait disturbance, abnormal movement patterns or weakness [21], cognitive symptoms as problems in concentration/learning difficulties or behavioral problems [21].

As disease progression is dominated by the deterioration of gross motor function, the standardized and validated Gross Motor Function Classification in MLD (GMFC-MLD-Score) was used to quantify the clinical course in these patients [20]. Using this measure, we have calculated the time frame in which patients of the juvenile cohort progress from first abnormalities in gross motor function (GMFC-MLD level 1) to loss of walking (GMFC-MLD level 3), in order to investigate the dynamic of disease progression.

For the dynamic of cognitive progression, two parameters were used: The time between onset of first symptoms and presentation of concentration problems and between onset of first symptoms and the start of language decline [21].

### MRI

In order to characterize disease-related MRI- the MRI-Severity Score was used to quantify cerebral changes. The score is based on a point system from 0 to 34 points that objectifies the extent of MRI-changes (involvement of the white matter as well as the occurrence of global brain atrophy) [35]. In addition, the pattern of involvement (more frontally pronounced vs more parieto-occipital dominance) was assessed visually.

### Genetic

The genetic analysis was done as part of the diagnostic procedure or as part of a research project as described in Böhringer et al. 2017 [7]. In order to analyze the relationship between genotype and age at onset, the most common combination of mutations was identified in our cohort.

### Statistical analysis

The similarity of clinical parameters between siblings was quantified using the Euclidian distance. The distance measures of the sibling pairs were compared to the Euclidian distances between all possible pairs from the (unrelated) patient cohort. The distances of the two groups (sibling pairs vs non-sibling pairs) were compared using the Mann–Whitney U test. The statistical evaluation, including descriptive statistics, was carried out using the software IBM SPSS Statistics, version 25.

## Data Availability

All relevant data are within the paper. Individual subject’s values from the whole patient cohort may be made available upon request addressed to the corresponding author, pending the approval of the Institutional Review Board of the University of Tuebingen, Germany.
